# Synthetic Degradable Polyvinyl Alcohol Polymer and Its Blends with Starch and Cellulose—A Comprehensive Overview

**DOI:** 10.3390/polym16101356

**Published:** 2024-05-10

**Authors:** Abdallah S. Elgharbawy, Abdel-Ghaffar M. El Demerdash, Wagih A. Sadik, Mosaad A. Kasaby, Ahmed H. Lotfy, Ahmed I. Osman

**Affiliations:** 1 Materials Science Department, Institute of Graduate Studies and Research (IGSR), Alexandria University, 163 Horrya Avenue, Shatby, P.O. Box 832, Alexandria 21526, Egypt; ab_pet_88@hotmail.com (A.S.E.);; 2 The Egyptian Ethylene and Derivatives Company (Ethydco), Alexandria 21544, Egypt; 3 School of Chemistry and Chemical Engineering, Queen’s University Belfast, Belfast BT9 5AG, Northern Ireland, UK

**Keywords:** degradable polymer, polyvinyl alcohol, biopolymer, starch, cellulose

## Abstract

Approximately 50% of global plastic wastes are produced from plastic packaging, a substantial amount of which is disposed of within a few minutes of its use. Although many plastic types are designed for single use, they are not always disposable. It is now widely acknowledged that the production and disposal of plastics have led to a plethora of negative consequences, including the contamination of both groundwater and soil resources and the deterioration of human health. The undeniable impact of excessive plastic manufacturing and waste generation on the global plastic pollution crisis has been well documented. Therefore, degradable polymers are a crucial solution to the problem of the non-degradation of plastic wastes. The disadvantage of degradable polymers is their high cost, so blending them with natural polymers will reduce the cost of final products and maximize their degradation rate, making degradable polymers competitive with industrial polymers that are currently in use daily. In this work, we will delineate various degradable polymers, including polycaprolactone, starch, and cellulose. Furthermore, we will elucidate several aspects of polyvinyl alcohol (PVA) and its blends with natural polymers to show the effects of adding natural polymers on PVA properties. This paper will study cost-effective and ecologically acceptable polymers by combining inexpensive natural polymers with readily accessible biodegradable polymers such as polyvinyl alcohol (PVA).

## 1. Introduction

A single-use plastic product is defined as a product that is made entirely or partially of plastic and is not intended, designed, or marketed to fulfill many trips or rotational motions during its lifespan by being sent back to the manufacturer for refilling or repurposing for the original purpose. Plastic trash can have significant worldwide effects on both the environment and human health. Reusable plastic products have a lower likelihood of ending up in the ocean than single-use products. Single-use plastic products that are most frequently seen on beaches in Europe, coupled with fishing gear, account for 70% of all marine trash in the EU [1]. According to Plastics Europe, a member of the European Trade Association, the amount of plastic generated globally has increased significantly annually. Two million tons was generated in 1950, and in 2021 almost 390 million tons was generated [2].

**Figure 1:**
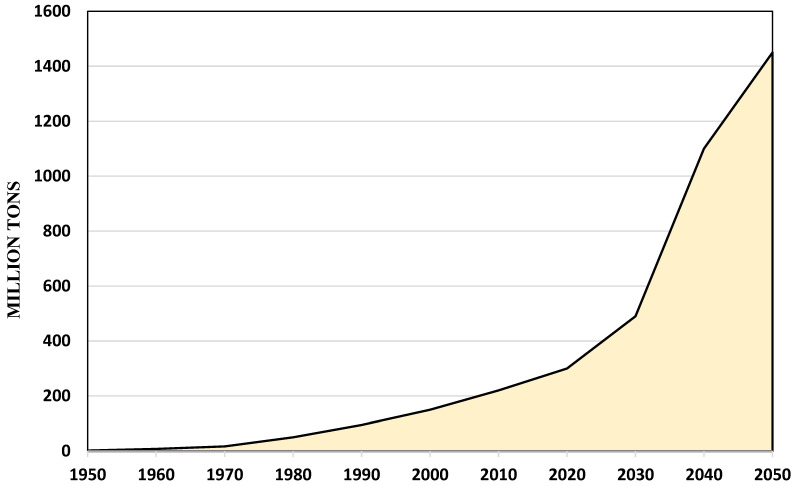
The expected quantity of plastic production per decade from 1950 to 2050.

After 2000, more than half of the entire amount of manufactured plastic was introduced into the market. It is anticipated that by 2050, production will increase to approximately 1480 million tons, a fourfold increase from the 2019 values. This is nearly three times the weight of all the people on the planet [3]. Figure 1 shows the expected global quantity of plastic produced per decade from 1950 to 2050 [3].

Despite the widespread usage of plastic consumer goods, especially single-use plastics, their current manufacturing and use are unsustainable [2,3,4,5]. To determine whether a material has a high consumption rate and the composition of the materials that are used in single-use plastic applications, it is crucial to identify the primary plastic raw materials utilized in these applications. The three primary materials used in single-use plastic products are polyethylene (PE), polyethylene terephthalate (PET), polystyrene (PS), and polypropylene (PP). PE is mostly utilized in plastic bags and agricultural items, whereas PET is a common material in bottles and PP is used in food/kitchen and cosmetic/detergent packaging products. Compared to PP, PS is utilized in comparatively tiny volumes in food and kitchen packaging items [6].

Plastic production began to boom in the 1940s and 1950s owing to the rapid rise in industrialization, and by 2020, it was predicted that global annual production would reach a total of 367 million metric tons. However, compared to the prior year, plastic manufacturing fell by roughly 0.3% because of the coronavirus (COVID-19) pandemic [7]. The amount of plastic produced worldwide in 2019 was the equivalent of 368 million metric tons (Mt) [8]; however, this figure is anticipated to double in 20 years [9]. Since plastics have a life cycle that now threatens planetary boundaries, pollution from plastics may surpass a predetermined threshold and become extremely critical, having a permanent worldwide influence on the atmosphere, ecosystems, and biodiversity levels [10]. It has been found that most of the plastic waste that enters the oceans contains hazardous bacteria, viruses, and microbial species that easily transmit toxic substances that eventually alter genetic diversity and affect ecosystems [10].

In 2016, aquatic habitats around the world received 19–23 million tons (Mt) of plastic waste; by 2030, that figure is anticipated to increase to 53 Mt annually [2]. By 2040, there is a chance that the amount of plastic waste in the ocean could increase to an estimated 30 million tons per year if prevention or damage-control efforts are not implemented, which would worsen the environmental impact [11]. By 2050, it is anticipated that practically all seabird species on earth could begin eating plastic garbage [12]. Approximately 14 million tons of plastic enter the ocean each year, primarily from Asian coastal regions. Over time, the lethal effects of plastic pollution have an influence on an estimated 700 species of aquatic life [13]. 

Mismanaged plastic wastes may plug drainages and rivers, causing flooding, mosquito breeding grounds, and the growth of disease-carrying flies and pests [14]. Heavy metals, plasticizers, and other manufacturing-related additives, as well as compounds that adsorb plastic from the environment, such as heavy metals, might all be effective delivery methods for harmful contaminants [15]. There is evidence that some microplastics have elements that are known to be mutagens, carcinogens, and reproductive poisons [16].

The effects of plastic on food webs are not yet fully known, although these compounds may be consumed at numerous trophic levels and bioaccumulate up the food chain [17]. Humans eat between 39,000 and 52,000 microplastic particles annually only from food and drink [18]. Plastics carry germs or parasites that may be on the plastics as well as additives from the production process and chemicals that have been adsorbed to the plastics as they reach the human food chain [19]. The demand for reasonably priced, long-lasting materials that provide convenience and improved usefulness is what is causing the tremendous rise in plastic manufacturing worldwide. Modern cultures are heavily reliant on plastic, and it is frequently utilized to create numerous everyday objects, including clothing, car interiors, and food and product packaging [20]. Despite its advantages, plastic packaging quickly produces plastic trash and, if handled improperly, can leak into the environment, harming both humans and ecosystems. According to research, plastics harm marine ecosystems like coral reefs by blocking light, entangling branching corals, seeping dangerous chemicals, and introducing alien biota [21]. 

Waste treatment systems have had a difficult time keeping up with the annual increase in the volume of plastic waste [8]. Many nations have been unable to consistently recycle significant amounts of plastic garbage due to a shortage of facilities for recycling and high purity criteria for reuse [22]. Globally, only 9% of plastic garbage has so far been recycled, 12% has been burned, and 79% of the remainder has been built up in ecosystems [23]. Most plastic waste is recycled, dumped in landfills, burned, or exported [24].

Recycling plastic waste can be used to make useful items like toys and bags [25]. The overall state of the environment has degraded in part due to plastics. To avoid the pollution’s adverse advertising effects, it is crucial to implement circular economy policies through recycling domestic waste [26]. When waste is polluted with green waste particles, mechanical recycling might only grow to be challenging and complex [27]. In a traditional mechanical recycling process, trash is collected, separated, cleaned, crushed, and pelletized for necessary conversion and reprocessing, which leads to the development of new goods without changing the chemical makeup of the material [28].

Polymers can be obtained from biomass wastes, such as wastes from plants, forests, biological industrial processes, municipal solid trash, algae, and animals. Pyrolysis is a mature and promising method for converting biomass-derived polymers into useful biochars. These products can be widely used in various fields, including carbon sequestration, power generation, environmental remediation, and energy storage. With an abundance of sources, affordability, and unique qualities, biochar made from biological polymeric materials shows much promise as a substitute electrode material for high-performance supercapacitors. A major challenge will be producing high-quality biochar to increase the application’s scope [29].

The process of gasification involves melting plastics in oxygen at 1200–1500 °C to create usable energy from waste. Pyrolysis is a process that includes heating plastics in an oxygen-free environment until the plastic waste disintegrates into gas and oil. All plastic polymers are broken down into very little molecules through this process.

Another method for getting rid of plastic waste is open landfilling, which is more of an environmental threat than carbon dioxide by around 23 times, and an estimated 150 million metric tons of plastic bottles ultimately end their cycle in landfills. More than half of greenhouse gas emissions have been estimated to come from gases produced in landfills. However, landfilling continues to be the approach to solid waste management that is most widely used worldwide. A plastic bottle can break down into microplastics in the ocean, where they can last for more than 500 years, and they have an expected lifespan of 500 years before they totally degrade in any landfill [30].

Some have suggested burning plastic to get rid of it, but burning it leads to the release of inhaled pollutants that have negative effects on the skin, eyes, human body, and cardiovascular health and cause headaches and nausea that may harm the nervous and female reproductive systems [11]. When plastics are burned in an open flame, most of the compounds that give them their distinctive characteristics, such as hardness, durability, malleability, color, and plasticity, are released into the atmosphere. These include several airborne toxins that have negative effects on human health, as previously stated [31]. Using incineration to manage plastic garbage has negative consequences on the environment since it releases highly hazardous compounds that eventually contaminate the air [32]. Table 1 summarizes the different types of plastic waste elimination processes. 

Table 1 proves that the different types of processes used to eliminate plastic wastes are not the most efficient way to decrease plastic wastes due to their disadvantages. Therefore, the optimum solution for managing plastic wastes is using degradable polymers which decompose naturally by bacterial activities after serving their purpose to produce natural byproducts, such as gases (CO_2_ and N_2_), water, biomass, and inorganic salts. 

**Figure 2:**
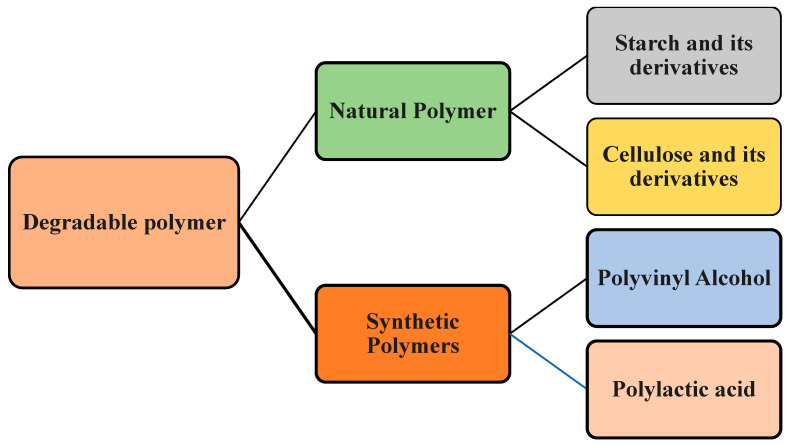
Different types of degradable polymer.

In this paper, we are going to show the differences between biopolymers and degradable polymers and summarize different types of degradable polymers, focusing on polyvinyl alcohol (PVA), its application, blends, and biodegradability. We also investigate the blending of different natural polymers, such as starch and cellulose, with PVA and their effect on the mechanical and structural properties of PVA. Also, we show the improvement in biodegradation properties that results from the addition of natural polymers to PVA. Figure 2 shows the different types of degradable polymers and blends that will be discussed in this paper. Natural polymers are polymers made from natural resources, while synthetic types are polymers made from industrial processes. 

## 2. Biodegradable Polymers and Biopolymers

Although biodegradable and biopolymers are two separate kinds of polymers, there is still confusion about the distinction between the two. Table 2 summarizes the differences between them.

After revealing the differences between biopolymers and degradable polymers, we will give examples of a few different kinds of biodegradable polymers and how they mix with other polymers. 

## 3. Polylactic Acid

One of the most well-known thermoplastic polyester biopolymers, PLA, has the properties of being biocompatible, biodegradable, and resorbable. PLA is a member of the aliphatic polyester family and is typically produced using hydroxy acid [41]. Using a vacuum-sealed heating method and lactic acid (LA), PLA was first created in 1932. Low-molecular-weight PLA is produced using this technique. Scientists have paid a lot of attention to PLA because of its many applications and non-toxic makeup [42].

PLA is also reasonably priced, and several industries have taken notice of its exceptional qualities. However, it is crucial to be aware of its structural restrictions, including its fragility, alterations, and functional changes [43]. There are two main ways to make PLA, a type of thermoplastic polyester with the chemical formula (C_3_H_4_O_2_)_n_: directly poly-condensing lactic acid and ring-opening polymerizing lactides [44,45]. At room temperature, PLA is insoluble in water and has unsubstituted hydrocarbons, but at higher temperatures, it immediately transforms into lactic acid in water-based solutions [45,46,47]. The capacity of PLA to distribute itself through carrier fluids depends on its density. Commercial PLA particles typically have a specific gravity of 1.24 to 1.25 [48]. The transition temperature of glass (Tg) and the melting temperature (Tm) are frequently used as indicators of the thermophysical characteristics of PLA. The usual range for the glass temperature of the transition Tg of PLA is 323–353 K [48]. The melting temperature (Tm) of PLA is typically between 393 and 453 K; however, it occasionally reaches 483 K [44,45]. According to reports, semicrystalline PLA possesses tensile strengths between 50 and 70 MPa, tensile moduli of 3 GPa, flexural strengths between 100 MPa and 5 GPa, and a length increase at the break of around 4% [49,50,51]. Depending on the application, PLA diverters can have a wide range of shapes and geometries. Powders, beads, flakes, particles (with varying roundness and sphericity), and fibers are some examples [48].

## 4. Polyvinyl Alcohol

PVA is an organic substance that is frequently found in tasteless and odorless powders or particles. It has great biocompatibility and hydrophilicity and stable chemical characteristics. PVA is primarily made by hydrolyzing polyvinyl acetate and substituting the hydroxyl group for an acetate group. PVA, with various levels of hydrolysis, can be manufactured by managing the hydrolysis stage [52,53]. PVA is produced commercially by hydrolyzing hydrophobic polyvinyl acetate since vinyl alcohol cannot be directly radically polymerized due to the unstable nature of the monomer [54].

It is important to note that the long-term storage of the solution, even at room temperature, may result in the formation of visible strands and slight turbidity, which are indicative of crystallization and the beginning of gelation. This is because PVA has a strong tendency to crystallize in a solution state, particularly when the degree of hydrolysis and concentration are high. As a result, storing concentrated solutions for an extended period (>15 wt%) can produce weak gels that do not meet the requirements of many applications. As a result, it is advised that a solution be incubated at a high temperature (>60 °C) for a few hours after long-term storage to disturb weak crystalline regions and restore the uniformity of the solution [55].

Due to PVA films’ very high cost and slow rate of biodegradation, researchers have concentrated more on enhancing their qualities over the past ten years by combining them with other environmentally acceptable biopolymers of various types and in various amounts. All the polymers and biopolymers employed were considered; however, chitosan, carboxymethyl cellulose (CMC), and starch were given more consideration for combining with PVA films due to the structure of their molecules and the presence of -OH functional groups [56].

There have been numerous efforts to replace synthetic polymers with cost-effective, biodegradable, renewable, and sustainable materials. These materials primarily consist of synthetic biopolymers produced chemically, such as polyvinyl alcohol (PVA), polycaprolactone, and polybutylene succinate; synthetic biopolymers produced by microorganisms, such as polyhydroxy-butyrate (PHB) and polyhydroxy-valerate; and naturally occurring biopolymers, such as starch, cellulose, chitosan, agar, gelatin, and alginate, among others; as well as mixtures [57].

## 5. Starch

The semi-crystalline polymer known as starch has a hydrophilic character. Due to its low cost, lack of toxicity, high biodegradability, and ease of availability, it is one of the biopolymers that has received the most research for use in food packaging. In terms of structure, starch is a complicated branched polymer in which the branch points and D-glucose units are connected by (1–4) links [58]. 

Regarding the molar mass, amylose makes up 10–20% of starch, while amylopectin makes up 80–90%. However, depending on the source of the starch, different amounts of amylopectin and amylose are present. An increase in elongation and strength occurs as the amount of amylose in the starch rises. Under heat, starch is not stable. At 150 °C, its glucoside linkages start to break down, and above 250 °C, the starch granules collapse. Its usage in the food packaging sector is constrained by weak mechanical qualities, low thermal processability, and particularly poor resistance to moisture [58,59]. 

Starch is one of the many natural resources that could be used to create biodegradable polymers because it is biodegradable, renewable, and readily accessible. Using the right plasticizer, starch can be heated up to create thermoplastic starch. However, because of their stiffness, brittleness, and poor mechanical and thermal properties, films made entirely of starch are not appropriate for packing purposes [60].

## 6. Cellulose

In terms of structure, cellulose is a linear polymer made up of glucose, and the glucose units are connected by β(1→4) glycosidic connections, which enable the cellulose chains to form strong interchain hydrogen bonds [6,61]. Even though cellulose has a number of benefits, including a high thermal resistance, UV barrier capacity, and FDA-acquired GRAS status, its hydrophilic nature, poor vaporized water barrier properties, and limited long-term stability, along with its poor mechanical properties due to its sensitivity to moisture, limit its use in food packaging at the industrial level [62].

Insoluble microfibrils, crystalline, and amorphous structural areas are produced by the abundant hydrogen bonding in cellulose, giving it good tensile strength and endurance [63]. Every component of a plant contains cellulose. Bacterial cellulose (BC) is cellulose that is formed from bacteria, algae, and tunicates. BC is more crystalline than plant cellulose, has a higher degree of polymerization, and can absorb more water than plant cellulose (60–90% higher crystallinity) [63,64,65]. As cellulose contains reactive functional groups, it can be chemically altered to yield a variety of cellulose derivatives by processes including carboxymethylation, etherification, hydroxypropylation, etc. Due to its flexibility, toughness, and water resistance, cellulose derivatives such as cellulose acetate, carboxymethylcellulose, and hydroxymethylcellulose are regarded as significant sources of biomaterial-based food packaging. However, they are costly when used in large quantities [61]. By being transformed into nanocrystals, cellulose can potentially be used as a reinforcement in nanocomposite films [61], microfibrils [66], and nanofibrils [67]. Cellulose reinforcements have superior mechanical qualities that are on par with those of glass and carbon nanofibers.

## 7. Hydroxypropyl Methylcellulose

The term “hydroxypropyl methylcellulose” (HPMC) refers to a class of cellulose ethers in which one or more of the three hydroxyl groups found in the cellulose ring have been substituted. HPMC is a hydrophilic (water-soluble), biodegradable, and biocompatible polymer with numerous uses in drug delivery, skin care products, adhesives, glue coatings, agriculture, and textiles [68,69]. Both aqueous and non-aqueous solvents can be used with HPMC because it is soluble in polar organic solvents as well. It has special qualities that make it soluble in both warm and cold organic solvents. Compared to its rivals made of methylcellulose, HPMC has higher organo-solubility and thermo-plasticity. When heated, it turns into a gel at temperatures between 75 and 90 °C.

The temperature at which glasses transition to HPMC can be lowered to 40 °C by decreasing the molar substitution of the hydroxyl propyl group. From an aqueous solution, HPMC creates translucent and flexible films. Due to their resistance to oil migration, HPMC films, which are typically odorless and tasteless, can be used to reduce the absorption of oil from fried foods like French fries. HPMC is widely employed as a stabilizer, an emulsifier, a protective colloid, and a thickening agent in the food sector. HPMC is utilized as an initial ingredient for coatings that have a moderate degree of elasticity, transparency, resistance to grease and fat, and moderate moisture and oxygen barrier qualities. Additionally, it serves as a tablet matrix for prolonged release as a tablet binder. Due to HPMC’s outstanding biocompatibility and low toxicity, its prospective use in the biomedical area has garnered the interest of both scientists and academics [69].

## 8. Blending of PVA with Natural Polymers

To improve characteristics, streamline processes, or cut costs, polymer blending is becoming more and more crucial in packaging applications. A few of the qualities that can be obtained by blending include tailoring surface parameters such as the coefficient of friction, adding color, enhancing adhesion, increasing production, improving stability, and gaining easy-opening features. The process of blending is cost-effective, somewhat easy to understand, and uses easily accessible processing technologies. Polymer-based films typically have altered physicochemical characteristics in comparison to their individual constituents. However, combining materials presents a significant compatibility difficulty. Compatibility has a significant impact on characteristics, including crystallinity, morphology, melting point, and glass transition temperature. Rigidity, processability, degradation behavior, and barrier qualities are, in turn, determined by these properties. The solubility parameter can be used to forecast how well two polymers will blend. Theoretically, two polymers are mutually soluble if their solubility parameter values are equal. Immiscible polymers can, however, be made more compatible by adding reactive functional groups or ester groups or by chemical alterations [70]. Blending polymers is a useful approach for removing flaws. It has the potential to create biodegradable film composites with better qualities at a reasonable price [71,72].

### 8.1. Polyvinyl Alcohol and Starch

Table 3 describes the effects of different starch ratios and different additives on the properties of PVA-blended films.

Table 3 shows that the addition of starch to PVA improves the degradation in soil and influences the thermal properties such that they become better than those of neat PVA. On the other hand, it decreases the mechanical properties because the intermolecular structure of starch is weak and highly amorphous. These findings show that this blend has a limitation in applications of high strength.

### 8.2. Polyvinyl Alcohol and Cellulose Derivatives

There are several types of PVA and cellulose blends, such as carboxy methyl cellulose and hydroxypropyl methylcellulose.

#### 8.2.1. Polyvinyl Alcohol and Carboxy Methyl Cellulose

Table 4 describes the effects of different CMC ratios and different additives on the properties of PVA-blended films.

Table 4 shows that the addition of CMC to PVA improves biodegradation and water solubility, but it decreases the thermal properties of neat PVA. The addition of CMC also decreases the mechanical properties because the intermolecular structure of CMC is weaker than that of PVA. 

#### 8.2.2. Polyvinyl Alcohol and Hydroxypropyl Methylcellulose

Table 5 describes the effects of different HPMC ratios and different additives on the properties of PVA-blended films.

Table 5 shows that the addition of HPMC to PVA increases the tensile strength and the antioxidant and antibacterial activity, which supports using this blend in high-strength applications. 

### 8.3. Biodegradation of Polyvinyl Alcohol

Table 6 describes the impact of the soil burial test on the biodegradability of different types of biopolymer/polyvinyl alcohol-blended films.

The addition of starch, cellulose, or cellulose derivatives to PVA enhances the water solubility and degradation in the soil of PVA because of the highly hydrophilic nature of cellulose, increasing rapid bacterial diffusion and leading to an increased biodegradability rate. However, the mechanical properties of some blends have decreased below those of pure PVA because of the high degree of amorphous and weak intermolecular forces. 

Any chemical, physical, or biological reaction that breaks covalent bonds in a polymer backbone and causes changes in its chemical structure and molecular weight is referred to as polymer degradation. The breakdown process of a polymeric artifact is propagated by reactive species or free radicals, which are created when the primary chemical bonds in the main or side chain break. Abiotic elements, such as heat, light, radiation, humidity, medium pH, mechanical stress, and chemical attack, initiate the polymer degradation process for these types of initiations to disrupt the chemical connections inside the polymer, an activation energy is required, when the localized energy of a chemical bond exceeds the overall energy of the bond, the bond breaks, a process known as chain scission. Polymer degradation results from the breaking of a more unstable bond positioned inside groups or short branches. This bond breakage might cause the side group to be lost or modified by the insertion of additional atoms such as oxygen [34].

Local variations in biodiversity and the presence of microorganisms are examples of extrinsic circumstances that influence a polymer’s degradation process, in addition to its intrinsic qualities. Consequently, the breakdown of materials can be broadly categorized as either biotic or abiotic (algae, bacteria, fungi, and radiation). Organic matter can break down in the natural world owing to a combination of biotic and abiotic factors. This is because certain microorganisms release extracellular enzymes that directly affect polymers; hence, prior fragmentation and molar mass reduction of the material are not required to make the microorganisms available [114]. Table 7 outlines the enzyme types that break down natural polymers, such as starch and cellulose.

**Figure 3:**
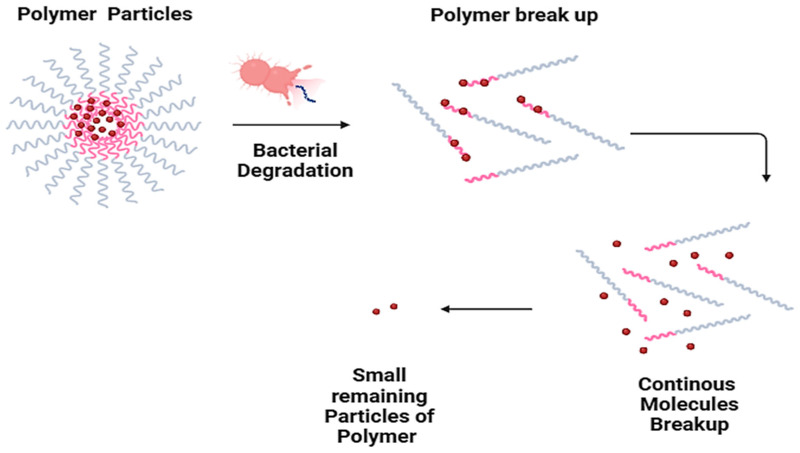
Polymer degradation mechanism.

As shown in Figure 3, the main biodegradation mechanism involves microorganisms adhering to the polymer surface and then colonizing the exposed surface. Following colonization, the polymer is hydrolytically broken down by enzymes released by bacteria, resulting in low-molecular-weight molecules until the final mineralization in CO_2_ and H_2_O [115].

## 9. Applications

**Figure 4:**
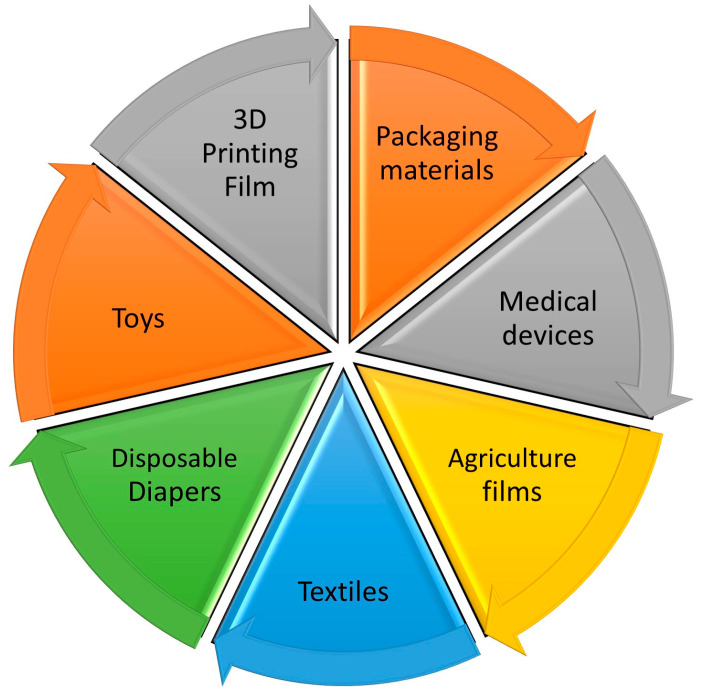
Different applications for PVA and natural polymer blends.

The goal of this research is to reveal the different applications of degradable polymers which are inexpensive and natural. Some applications of the films produced are listed in Figure 4.

A starch/PVA blend can be used as a biodegradable film in packaging to lessen its impact on the environment. PVA/starch or PVA/HPMC blend sheets can be used as burial films in agriculture. These coatings aid in controlling temperature and retaining soil moisture. They do not need to be removed after their function is fulfilled since they can naturally deteriorate. The textile industry may employ starch/PVA mixed films to make items like non-woven textiles and throwaway apparel. These products can be made to decompose organically once they have been used.

Certain disposable diapers can be made of biodegradable materials, such as the PVA/HPMC blend, which lessens the harm that regular disposable diapers do to the environment.

## 10. Conclusions

Plastic trash disposal is currently a significant environmental issue. Plastics are used more frequently and in diverse ways in our daily lives, which influences on the environment. Due to the carbon dioxide emissions caused by the burning of typical non-biodegradable polymers, such as polyethylene, polypropylene, and polyvinyl chloride, there is an increasing concern regarding global warming. The best way to manage non-biodegradable plastic waste is to switch to biodegradable polymers because they are more cost-effective for recycling or reuse than non-biodegradable materials. Many different types of degradable polymers, such as polylactic acid, polycaprolactone, and polyvinyl alcohol, with natural polymers, such as starch and cellulose, have competitive specifications compared to non-degradable polymers. There is great interest in biodegradable polymers for short-term use in fields such as surgery, pharmacology, agriculture, and the environment.

Our findings prove that using inexpensive natural polymers with a commercially available biodegradable polymer such as PVA to generate ecologically acceptable, cost-effective films would make the polymers more affordable while simultaneously improving their thermal characteristics and rates of deterioration, giving them a serious competitive advantage over industrial polymers that are widely used today.

## Figures and Tables

**Table 1:** The different types of plastic waste elimination processes.

Process	Definition	Advantages/Disadvantages
Traditional recycling	Plastic waste can be used to make useful items, such as toys and bags [1]	Traditional recycling does not require a complicated process and does not emit greenhouse gases, but only requires creative people to convert plastic into simple things like toys and bags
Traditional mechanical recycling	Type of recycling in which plastic trash is collected, separated, cleaned, crushed, and pelletized for necessary conversion and reprocessing, which leads to the development of new goods without changing the chemical properties of the material [2]	Does not change the chemical behavior of plastic Costly in comparison to traditional recycling In the palletization process, gases emit CO, which has a negative effect on the environment
Chemical recycling	Chemical processes are used in the breakdown of polymers, with methanolysis and glycolysis being the most widely used Chemical recycling involves gasification and pyrolysis The most widely used method is pyrolysis, which is the procedure for crushing and melting sorted plastics to produce usable diesel and other petrochemical chemicals [3]	Changes the chemical behavior of the component Costly in comparison to traditional recycling and traditional mechanical recycling During this process, greenhouse gases are emitted, which have a negative effect on the environment, especially weather
Gasification (chemical recycling)	The process involves melting plastics in oxygen at 1200–1500 °C to create usable energy from waste	Changes the chemical behavior of the component Costly During this process, greenhouse gases are emitted, which have a negative effect on the environment, especially weather
Pyrolysis (chemical recycling)	The Process involves heating plastics in an oxygen-free environment until plastic waste disintegrates into gas and oil. All plastic polymers are broken down into very small molecules through this process	Changes in the chemical behavior of the component. Costly During this process, greenhouse gases are emitted, which have a negative effect on the environment, especially weather
Landfilling	A process by which plastic waste is buried in land to get rid of it Ultimately, 150 million plastic bottles end in landfills [4]	Creates more of an environmental threat than carbon dioxide by around 23 times [4] More than half of greenhouse gas emissions have been estimated to come from gases produced at landfills [4]
Burning plastic	The process by which plastics are burned in an open flame, and most of the compounds that give them their distinctive characteristics, such as hardness, durability, malleability, color, and plasticity, are released into the atmosphere	This leads to the release of inhaled pollutants that have negative effects on the skin, eyes, human body, and cardiovascular health and cause headaches and nausea that may harm the nervous and female systems [5] Releases several airborne toxins that have negative effects on human skin, eyes, and heart health and cause headaches and nausea that may harm our nervous and reproductive systems [6] Using incineration to manage plastic garbage has negative consequences for the environment since it releases highly hazardous compounds that eventually contaminate the air [7]

**Table 2:** The differences between biodegradable polymers and biopolymers.

Type	Biodegradable Polymers	Biopolymers
Definition	Organic material degrades by biological activity, primarily the enzymatic action of microbes. According to the European Standard EN 13432-2000 [33], the final products are CO_2_, fresh biomass, and water under aerobic circumstances (with oxygen present) or methane under anaerobic conditions (without oxygen present) [34]	Polymers are made from organic or plant-based materials, such as byproducts from agriculture, horticulture, and crops [35]
Examples	(A) Natural polymers, such as polysaccharides: starch–cellulose–chitosan and 2-protein and lipids [36] (B) Polymers obtained by microbial production: polyhydroxyalkanoates (PHAs) such as poly(hydroxybutyrate) (PHB) [36] (C) Polymers chemically synthesized using monomers obtained from agro-resources like PLA [36] (D) Polymers whose monomers and polymers are both obtained by chemical synthesis from fossil resources: polycaprolactones (PCLs), polyester amides (PEAs), polyvinyl alcohol (PVA) [36]	(A) Biopolymers made up of renewable raw materials (bio-based), which are biodegradable [37]: Synthetic polymers from renewable resources such as polylactic acid (PLA) [37] Biopolymers produced by microorganisms, such as PHAs Naturally occurring biopolymers, such as starch or proteins Biopolymers made from renewable raw materials (bio-based), which are not biodegradable [37]: Renewable resource synthetic polymers, such as specific polyamides from castor oil (polyamide 11), specific polyesters based on bio-propanediol, bio-polyethylene (bio-LDPE, bio-HDPE), bio-polypropylene (bio-PP), bio-polyvinyl chloride (bio-PVC) based on bioethanol (e.g., from sugarcane), etc. Naturally occurring biopolymers such as natural rubber and amber (B) Biopolymers made from fossil fuels which are biodegradable [38]: Made from crude oil or natural gas and are certified biodegrade-blend compostable. Polyvinyl alcohol (PVA) PCL, polybutylene succinate (PBS), and certain “aliphatic–aromatic” polyesters are at least partly fossil-fuel-based polymers, but microorganisms can degrade them
Advantages	Have the least detrimental impact on the environment in terms of pollution Renewable [35] Affordable [35] Cost-effective [39,40] Biocompatibility [39,40] Optimum option for various biomedical applications [39,40]	Have the least detrimental impact on the environment in terms of pollution Renewable [35] Affordable [35] Cost-effective [39,40] Biocompatibility [39,40] Optimum option for various biomedical applications [39,40]
Quantity produced	The overall quantity of bio-based polymers produced in 2020 was 4.2 million tons, or 1% of the total amount of fossil-fuel-based polymers produced. The CAGR is now, with 8%, much higher than the growth of polymers (3–4%) for the first time in a long time, and this trend is anticipated to last through 2025 [38]	

**Table 3:** The effects of different starch ratios and different additives on the properties of PVA-blended films.

Polymer Blend Type	Effect	Ref.
PVA/corn starch mixes designed for wood adhesives that are used with polyvinyl acetate (PVAc)-white glue. Glyoxal, boric acid, citric acid, and glutaraldehyde were used in low concentrations (0.1 wt%)	Improvement in the tensile shear strength Tensile strength of the bonded wood increased by 16% in the sample containing citric acid, 13% for glutaraldehyde, 8% for boric acid, and approximately 7% for glyoxal crosslinked wood adhesive Improved their thermomechanical qualities	[73]
Low-weight chitosan, polyvinyl alcohol, and maize starch; the PVA concentration ranged from 0 to 40 wt.%, while the St/Chit weight ratio was set at 70/30	PVA increased phase separation and mechanical properties and had a good biodegradation effect	[74]
Polyvinyl alcohol (PVOH)/starch ratios of 0–60%	Good material compatibility in all the compositions Improved the mechanical, gas permeability, and functional characteristics of the potato starch films	[75]
Polyvinyl alcohol (PVOH) mixed with pregelatinized starch (PSt) by incorporating antibacterial agents, such as dodecyl dipropylene triamine (TRIAMEEN) and 2-hydroxypropyl-3-piperazinylquinolinecarboxylic acid methacrylate (HPQM)	The mechanical and water-soluble qualities of the blend were not considerably affected by the addition of antibacterial agents to the blend film The blend films containing HPQM showed higher antibacterial activity than the films containing TRIAMEEN against both *Escherichia coli* (*E. coli*) and *Staphylococcus aureus* (*S. Aureus*)	[76]
Polyvinyl alcohol (PVA) and maize starch with the addition of purple sweet potato extracts (PSPE) and red cabbage extracts (RCE)	Low water vapor permeability of film With increasing extract content, there was a significant increase in thickness and mechanical and thermal properties Light transmittance was significantly decreased	[77]
St/PVA films produced using the blowing extrusion technique with a St/PVA ratio of 4:6	Fat-soluble liquid pesticides were facilitated by the dissolved St/PVA films containing amphiphilic groups The mechanism of pesticide–weed leaf surface interaction was postulated to explain the increased efficiency of pesticides packaged in the St/PVA films The technique of using PVA blending and MA-enhanced esterification to create starch-based films that are appropriate for pesticide inner packaging materials is effective	[78]
Starch, polyvinyl alcohol, and graphene oxide.	Good tensile strength, good thermal stability of the film, and good biodegradability rate The improvement in film moisture resistance increases with increased GO concentration	[79]
Nanocomposite films of polyvinyl alcohol, graphene oxide, starch, and silver (PVA/GO/Starch/Ag)	Good tensile strength High antibacterial characteristics Good thermal stability	[80]
Starch/polyvinyl alcohol (PVA) degradable straws using a twin-screw extrusion technique, with varying PVA concentrations	The starch/PVA straws with 40% PVA (PS4) had the highest starch and PVA dispersion uniformity The strongest hydrogen bonds between 40% polyvinyl alcohol and starch allowed for the strongest molecular interactions PS4’s mechanical qualities and water resistance were greatly enhanced by the strongest compatibility and molecular connections The swelling volume of PS4 decreased by 45.5% (4 °C) and 65.2% (70 °C) compared with that of the starch/PVA straw with 0% PVA (PS0). After soaking, the diameter strength increased by 638.7% (at 70 °C for 15 min) and 540.1% (4 °C for 1 h). The water absorption decreased by 45.3% at 4 °C for 30 min and 27.6% at 70 °C for 30 min	[81]
By using 88% hydrolyzed (PVA/cassava starches (NCS, HCS, and PCS), a mixture of the mixes was plasticized with glycerol or a glycerol–sorbitol mixture by solution-casting	The PVA/HCS and PVA/PCS blend films exhibited a PVA-rich/starch-rich bilayer structure when scanned using scanning electron microscopy The significant degree of swelling in the mixed films led to rapid degradation The discovered mixed films exhibited higher strength and elongation at break than low-density polyethylene	[82]
PVA/starch films with water extracts from basil leaves were added as antibacterial agents following the addition of basil leaf extracts	All films retained their tensile strengths and resembled neat PVA/starch films Excellent inhibition of the two tested pathogens, *S. aureus* and *E. coli*, was demonstrated by antimicrobial tests The freshness of chilies wrapped in basil extract-containing films was better maintained than that of unpacked ones The findings of this study indicate that these films can be effectively employed to package fragile fruits and vegetables	[83]
Corn starch (CS) and polyvinyl alcohol (PVA) matrix for a Pickering emulsion loaded with curcumin	Pickering emulsion that was loaded with curcumin turned from yellow to red over time Good mechanical properties, good barrier capabilities, and good antimicrobial activity	[84]
Lemon peel/polyvinyl alcohol/starch matrix	Powerful connection film Strong compatibility and bonding with the polymer matrix Good mechanical properties increase with increasing addition of peel	[85]
Polyvinyl alcohol (PVOH) and corn starch (ST) with a pineapple peel extract (PPE), with PPE concentrations of 5%, 10%, 15%, and 20%	Films’ thickness and water vapor permeability increased with increasing concentration of PPE Elongation at break improved by PPE concentration The thermal stability of all PPEs with integrated films was improved as breakdown occurred above 300 °C Good antioxidant capabilities of PVOH/ST films Good active biodegradable packaging materials	[86]
Anthocyanins or betacyanins or anthocyanin/betacyanin mixtures (in various weight ratios of 3:1, 1:1, and 1:3) in starch/polyvinyl alcohol (PVA) films	Anthocyanins are more pH-sensitive than betacyanins High compactness of the films Low crystallinity Good ability to block ultraviolet and visible light and water vapor and good antioxidant, good antibacterial, and ammonia-sensitive capabilities	[87]
Corn starch, polyvinyl alcohol (PVA), and glycerol that also contained polylysine	Polylysine reduced the strength of each crystal peak Good thermal stability of composite films As the polylysine concentration increases, the antibacterial power of the composite film increases Water solubility decreased as polylysine content increased Good mechanical, physical, and antibacterial properties and might find use as a unique antimicrobial packaging material	[88]
PVA/starch nanocomposite film reinforced with sugarcane bagasse cellulose nanofiber (CNF), with a varying ratio of 1–6 wt% of the cellulose nanofiber suspension applied to the PVA/starch film	When the CNF loading of SCB was 4 wt%, the thermal characteristics, tensile strength, and elongation at break of the PVA/starch/cellulose nanofiber improved The addition of cellulose nanofiber reinforcement to the PVA/starch film resulted in a 254% increase in tensile strength (85 MPa) This composite film made of PVA, starch, and CNF with lemongrass essential oil inhibited the growth of S. aureus, a Gram-positive bacterium	[89]
Intelligent packaging labels in which anthocyanin-rich extract was immobilized in starch/polyvinyl alcohol matrices	The label is highly crystalline The extracts improved labels’ UV-Vis light blocking, antioxidant, and pH-dependent color-changing properties The label containing haskap berry extract had the best light-blocking properties The labels containing blackberry or blueberry extract had the best antioxidant properties The labels with black eggplant peel extracts were appropriate for shrimp freshness monitoring The label with purple cabbage extract was appropriate for monitoring the freshness of pork	[90]
Polyvinyl alcohol/boiled rice starch blend film in the presence of solar irradiation and the addition of silver nanoparticles (PVA/BRS/sAgNPs)	Enhanced mechanical and optical capabilities with reduced water sensitivity Good antibacterial activity Good film biodegradability	[91]
Hydrolyzed starch (HST)/pregelatinized starch (PST) at different concentrations with 20% glycerol	Blend with HST had less overall thermal degradation The elongation at break was reduced by a factor of two, while the tensile strength rose after blending with PST Good water solubility for both films	[92]
Starch/polyvinyl alcohol (PVA) straws with varying PVA contents	Starch/PVA straws with 40% PVA (PS4) exhibited the best compatibility Good mechanical properties and water resistance	[81]
Starch/PVA (10, 30, 50, 70, and 90% of the starch weight) composite films	The mechanical and barrier qualities of composite films were enhanced by increasing the addition of PVA to starch polymer from 10 to 90 The WVP and OP of composite films decreased Good film barrier against water vapor and oxygen gas Good biodegradability rate	[93]

**Table 4:** The effects of different CMC ratios and different additives on the properties of PVA-blended films.

Polymer Blend	Effect	Ref.
Polyvinyl alcohol (PVA), sodium carboxymethylcellulose (CMC), and N-(2-hydroxyl) propyl-3-trimethylammonium chitosan chloride (HTCC)	The addition of CMC improved the mixed films’ strength and flexibility The use of CMC produced an improved swelling ratio, proper moisture permeability, and increased water absorption capacity Good antibacterial efficacy Suitable for use as biomaterials in medical applications	[94]
Carboxymethyl cellulose and polyvinyl alcohol (CMC/PVA)-based hybrid polymer (HPe) system with different ratios of composition	The mix of 80:20 compositions of the CMC/PVA HPe system was the ideal ratio, increasing the conductivity of the substance by an order of magnitude Lowest levels of crystallinity	[95]
CMC/PVA/CuO bio-nanocomposites for covering processed cheese	Film’s gas transmission rate (GTR) and water vapor transmission rate (WVTR) were reduced by the addition of CuO-NPs Good antimicrobial activity A Strong contender for applications in cheese packaging	[96]
Polyvinyl alcohol (PVOH), clove oil, and carboxymethyl cellulose (CMC)	Tensile strength and puncture resistance were both increased with an increase in PVOH concentration The rate of water vapor transmission was reduced. Good antibacterial activity, so it could be used as active packaging material for meat preservation	[97]
Carboxymethyl cellulose (CMC)/polyvinyl alcohol (PVA) film emulsified with oleic acid (OL) and mixed with rosemary essential oil (REO) at various concentrations of REO (0.5, 1.5, and 3%)	The UV absorbance and elongation at the break of the films increased. The films’ tensile strength and thermal stability decreased Strong antibacterial and antioxidant effects were observed in films of different concentrations	[98]
Polyvinyl alcohol (PVA) and carboxymethyl cellulose (CMC)	The antifungal and antioxidant qualities of the produced films were improved. The films showed a strong UV inhibitory impact. The CEO Pickering emulsion proved a perfect substitute to incorporate with CMC-PVA-based films	[99]
Cellulose, glycerol, and polyvinyl alcohol	Polyvinyl alcohol increases film toughness and film thickness As glycerol concentration increases, toughness falls, and it provides a UV protective effect in the films. The films have ideal transparency values	[100]

**Table 5:** The effects of different HPMC ratios and different additives on the properties of PVA-blended films.

Polymer Blend	Effect	Ref.
Polyvinyl alcohol (PVA)/hydroxypropyl methylcellulose (HPMC) film matrix to immobilize roselle anthocyanin extract (RAE)	RAE was strongly immobilized in the PVA/HPMC matrix Hydrophobic properties significantly decreased, and film thickness increased Tensile strength increased High antioxidant and antibacterial activity Improved the functional qualities and freshness monitoring impacts of PHR films	[101]
Polyvinyl alcohol (PVA) modified with hydroxypropyl methylcellulose (HPMC)	The films with a ratio of 3:1 for HPMC and PVA had higher tensile strength The films have good physical properties The addition of HPMC enhances the functional properties of films by altering their chemical structure and creating novel interactions	[102]
Hydroxypropylmethylcellulose (HPMC) to prepare thin films containing up to 20% cannabidiol (CBD). Soft and flexible polyvinyl alcohol (PVA) was used as the supporting layer	The bilayer film exhibited exceptional mechanical characteristics, with an elongation of 30% and a tensile strength of 8.54 N/mm^2^ PVA backing layer not only enhances the mechanical characteristics but also extends the duration of film disintegration by up to 90 min The HPMC-PVA bilayer offers mucoadhesive qualities for oral administration and regulates release for increased absorption of cannabidiol, making it an appropriate polymer matrix for cannabidiol delivery	[103]
Chitosan/hydroxypropyl methylcellulose/polyvinyl alcohol mix film (CHP) with modified bamboo fiber treated with coupling agent	The film’s WVP and water solubility were both reduced by 41.63% and 36.93%, increasing its waterproof capacity Both the elongation at break and the tensile strength rose by 61.97% and 8.07%, respectively	[104]
Povidone-iodine (PVP-I)-integrated polyvinyl alcohol-hydroxypropyl methylcellulose (PVA/HPMC_B)-based film	When PVP-I and PVA/HPMC_B films were utilized, the inflammatory stage was diminished, and the dermal “scar” was less PVP-I integrated into PVA-hydroxypropyl methylcellulose film is a promising medication carrier, acting more quickly and successfully than the other two dressing materials considered. These innovations offer a possible substitute for tissue regeneration and wound healing	[105]
HPMC and PVA ODF (orally dissolving film) (HPMC: PVA) with ratios of F1 (3:0), F2 (2:1), F3 (1.5:1.5), F4 (1:2), and F5 (0:3)	The use of a single HPMC produces films with rough surfaces Using a single PVA produces sticky films Using a single HPMC and PVA together produces smooth, non-sticky films	[106]
Polyvinyl alcohol/hydroxypropyl methylcellulose/chitosan blend film (CHP) using bamboo fiber	The film created with 1 mL of coupling agent had the best overall performance The WVP and water solubility of the film were reduced by 36.93% and 41.63%, respectively, thereby increasing its waterproof capacity There was an enhancement of 8.07% in the elongation at break and 61.97% in the tensile strength	[104]

**Table 6:** The impact of the soil burial test on the biodegradability of different types of biopolymer/polyvinyl alcohol-blended films.

Polymer Blend	Effect	Ref.
Chitosan/cassava starch/PVA	The best biodegradable plastic tested decomposed by 50.45% of its weight after 30 days of being buried in the ground	[107]
Different amounts of cellulosic fibers in the form of powder (water hyacinth powder, “WHp”) were added to the topical starch (TS)/PVA blend	All of the TS/PVA/WHp films can biodegrade. Adding WHp at a rate of 20% *w*/*w* greatly improves the films’ stability against moisture while retaining useful elasticity for a variety of applications	[108]
Octenyl succinic anhydride (OSA) esterified potato starch, gliadin, and polyvinyl alcohol (PVA)	All composite plastics disintegrated quickly for the first 20 days, then gradually over the next 56 days The addition of gliadin and OSA starch increased the biodegradability. The disintegration rate of composite plastics reached its greatest level of 94.6% at 21 days when the ratio of OSA starch/gliadin was 2:2 and the replacement ratio of PVA was 75%	[82]
PVA (10, 30, 50, 70, and 90% of the starch weight per gram)	All three tested film samples were shown to decay quickly in soil, with extremely substantial weight loss Pure starch films degraded completely in one week, while pure PVA and SP70 composite films took three weeks to degrade completely Samples fully deteriorated after 21 days, revealing no visible signs of any film fragments The outcomes showed that the composite film’s deterioration rate was delayed by combining PVA and starch	[93]
Polyvinyl alcohol (PVA) starch (S) in the presence of glacial acetic acid as a crosslinking agent	Depending on the Mw of PVA, PVA/S-blend films degraded between 10 and 14 days in dry soil PVA (205 × 10^3^ g/mole)-blend films degraded in 13–14 days, and PVA (31 × 10^3^ g/mole)/S-blend films did so in just 10 days It is evident that increasing the molecular weight of PVA lengthens the time it takes for PVA/S mixed films to biodegrade in dry soil These findings show that the addition of S to PVA improves the polymer matrix’s ability to degrade through soil	[109]
Cellulosic material barley husk (BH) and PVA (polyvinyl alcohol)/starch and starch-based composite sheets for packaging applications	The rate at which the films degraded in soil was accelerated by the addition of starch to PVA Crosslinking of the polymer matrix led to a reduction in the biodegradation of the film After 120 days of soil burial, the composite film (PVA/0.5 C Starch-1BH) demonstrated a 33% weight decrease Due to decreased hydrophilicity, the grafted BH composite film demonstrated lower weight loss	[110]
Modified maize starch combined with PVA in various ratios	The addition of modified starch increases its ability to absorb water The mix lost the most weight after enzymatic breakdown when 20% of modified starch was added The inclusion of the starch was found to improve the degradability due to the growth rate of the microorganism	[111]
Neat polyvinyl alcohol (PVA)/starch (ST)/glycerol (GL)/halloysite nanotube (HNT) nanocomposite films with different HNT contents	After 24 weeks, biodegradation studies demonstrated that PVA/ST, PVA/ST/GL blends, and PVA/ST/GL/HNT nanocomposite films shrank in size and were supposed to become more fragile and wrinkly films Significant microbial activity due to rising temperatures increases oxygen availability The degradation process proceeded at a rather modest rate After 24 weeks, neat PVA films exhibited the slowest biodegradation rate of all the materials, 5.87%, indicating that PVA had great resistance to biodegradation in soil	[112]
PVA/starch and citric acid as plasticizing agent (buried for 120 days in a pot of farm soil)	Films ultimately shrank in size and appeared brittle and hard St/PVA-crosslinked film and fiber-reinforced St/PVA composite blend films both experienced weight loss degradation of 34.54% and 45.65%, respectively Crosslinking causes the film to deteriorate in soil at a slow rate	[113]
Maize starch/chitosan composite film	After composting for 60 days, the films started to noticeably disintegrate The sample weight losses were monitored every 20 days The St/Chit film showed the fastest rate of deterioration because it was nearly destroyed after 20 days of testing The St/Chit/PVA films were substantially degraded by the time the soil burial test was over	[74]

**Table 7:** Types of enzymes/bacteria that break down natural polymers (starch and cellulose) [114].

Type of Biodegrading Enzyme/Bacteria	Polymer Type	Biodegradation Mechanism	Mode of Action and Mechanism
Amylase	Starch	Hydrolysis	Breaks down the α-1,4-glycosidic bonds in starch, producing glucose
Cellulases	Cellulose	Hydrolysis	Breaks down the β-1,4-glycosidic bonds in cellulose, producing glucose

## Data Availability

The data used to support the findings of this study are included within the article.
